# Protective Lifestyle Changes in Family Homozygous for APOE4

**DOI:** 10.1002/alz70858_098134

**Published:** 2025-12-24

**Authors:** Charlotte Horowitz, Eugene Pahk

**Affiliations:** ^1^ Western University of Health Sciences, Pomona, CA, USA; ^2^ Mission Community Hospital, Panorama City, CA, USA

## Abstract

**Background:**

Genetic factors play an important role in Alzheimer's Disease development. Specifically, the apolipoprotein E (APOE4) gene has the strongest associated risk factor for disease development and drastically increases risk in patients who are homozygous for the gene. APOE4 is involved in lipid metabolism and contributes to Alzheimer's by forming amyloid‐beta protein and neurofibrillary tangles in the brain. In terms of risk reduction, there are studies outlining various lifestyle modifications like specific diets and regular exercise.

**Method:**

Case Study

**Result:**

This case focuses on a 69‐year‐old female with new onset memory loss and genetic testing revealing her to be homozygous for the APOE4 gene. She has two older siblings who were both diagnosed with early onset Alzheimer's disease and who were also homozygous for the APOE4 gene. The siblings presented with clinically significant cognitive impairments at ages 62 and 64. When the current patient learned of her genetic status and watched her siblings' cognition deteriorate, she made major lifestyle changes to try and prevent the disease. The patient quit her job to focus on daily exercise, healthy sleep habits, and stress reduction. She additionally began following a vegan diet prioritizing fruits, vegetables, and healthy fats. Currently, the patient's clinical symptoms are progressing significantly slower in comparison to her siblings, and she has not yet been given an official diagnosis of Alzheimer's disease.

**Conclusion:**

Around 2% of the world's population is homozygous for APOE4, making it increasingly rare for three siblings within one family to present with this genetic makeup, two of whom go on to develop early onset Alzheimer's disease. Further medical testing is warranted regarding the current patient's cognitive status and biomarkers for Alzheimer's Disease. This case study could contribute to a greater understanding of protective lifestyle changes that delay Alzheimer's disease, specifically in patients who are homozygous for the APOE4 gene.

